# The association of Th17/Treg cells expression in peripheral blood and chronic spontaneous urticaria: A protocol of systematic review and meta-analysis

**DOI:** 10.1097/MD.0000000000022014

**Published:** 2020-09-04

**Authors:** Qianying Yu, Wenxia Lin, Jie Zhang, Li Peng, Qiaoqiao Kong, Rubin Zhong, Yan Lan, Min Xiao, Mingling Chen

**Affiliations:** a Department of Dermatology, Hospital of Chengdu University of Traditional Chinese Medicine; b Chengdu University of Traditional Chinese Medicine, Chengdu; c Department of Dermatology, Honghe Hani-Yi Autonomous Prefecture TCM Hospital, Honghe Hani-Yi Autonomous Prefecture, Yunnan Province; d Department of Dermatology, Guangzhou Zengcheng District Hospital of Traditional Chinese Medicine, Guangzhou, Guangdong Province; e Department of Dermatology, Jianyang Chinese Medicine Hospital, Jianyang, Sichuan Province, P.R. China.

**Keywords:** chronic spontaneous urticaria, systematic review, Th17/Treg

## Abstract

**Background:**

The pathogenesis of chronic spontaneous urticaria (CSU) is not clear, but its occurrence is closely related to the immune state of the body, that is, the balance of T cell subsets. Previous studies have confirmed that the dynamic imbalance of Th1/Th2 cells in CD4+T cell subsets of T cell subsets is closely related to the pathogenesis of CSU, but there are few studies on the relationship between the dynamic imbalance of Th17/Treg cells in CD4+T cell subsets and the pathogenesis of CSU. The purpose of this study is to evaluate the relationship between Th17/Treg cells expression in peripheral blood and CSU, so as to provide a reference basis for the pathogenesis of CSU.

**Methods:**

PubMed, Embase, CENTRAL, Web of Science, China Biology Medicine Database, China National Knowledge Database, Wan Fang Database, and Chongqing VIP Database will be searched to collect case-control studies and cohort studies evaluating the relationship between Th17/Treg cells expression in peripheral blood and CSU. The search time limits will be from the establishment of the database to December 2020. The meta-analysis will be carried out with the RevMan V.5.3 statistical software. The quality of all included studies will be evaluated by the Newcastle-Ottawa scale.

**Results:**

The results of this study will comprehensively evaluate the Th17/Treg cells expression levels in peripheral blood of patients with CSU, and provide a reference basis for the pathogenesis of CSU.

**Conclusion:**

The findings of this study may provide new evidence for the relationship between Th17/Treg cells balance in peripheral blood and CSU.

**OSF registration number:**

DOI 10.17605/OSF.IO/S8MYW

## 1 Introduction

Urticaria is a condition characterized by the development of wheals (hives), angioedema or both, including chronic spontaneous urticaria (CSU) and inducible urticaria.^[1]^ The CSU refers to those with spontaneous appearance of wheals, angioedema, or both for >6 weeks due to known or unknown causes. The incidence of CSU is 0.5% to 0.1%, the course of the disease is prolonged, and the recurrence rate is high, which seriously affects the quality of life of the patients and aggravates the economic burden of the patients.^[2]^

The pathogenesis of CSU is not clear, but its occurrence is closely related to the balance of T cell subsets, that is, the immune state of the body. In recent years, many studies have confirmed that the pathogenesis of CSU is closely related to the dynamic imbalance of Th1/Th2 cytokines,^[3–5]^ but there are few studies on the relationship between Th17/Treg immune imbalance and the pathogenesis of CSU. In the occurrence and development of most autoimmune diseases and inflammatory diseases, the destruction of Th17/Treg balance plays an important role,^[6]^ which is related to the occurrence of many autoimmune diseases, such as anaphylactoid purpura, atopic dermatitis, systemic lupus erythematosus, hashimoto thyroiditis, rheumatoid arthritis, and so on.^[7–11]^ The CSU is a multifactor autoimmune disease with the disorder of human autoimmune function as the core.^[12]^ The immune imbalance of Th17/Treg may play a key role in the pathogenesis of CSU.

## 2 Methods

### 2.1 Study registration and ethics

This protocol has been registered at the Open Science Framework (OSF). Original data will not be collected, and no ethical approval will be required. This protocol will adhere to the preferred reporting items for systematic reviews and meta-analysis protocols statement.^[13]^

### 2.2 Inclusion criteria

#### 2.2.1 Type of study

Case-control studies and cohort studies that evaluate the relationship between Th17/Treg cells expression in peripheral blood and CSU.

#### 2.2.2 Participants

Patients in the case group should be diagnosed as CSU, regardless of race, sex, age, and course of the disease. People in the control group should be without CSU.

#### 2.2.3 Exposure factors

The expression levels of Th17/Treg cells in peripheral blood will be regarded as exposure factors.

#### 2.2.4 Types of outcomes

The outcome measurements include the expression levels of Th17 and Treg cells in peripheral blood.

### 2.3 Exclusion criteria

The exclusion criteria will be as follows: duplicate literature, literature with incomplete data, and animal experiments.

### 2.4 Electronics searches search strategy

We will comprehensively search the literature sources of the PubMed, Embase, CENTRAL, Web of Science, China Biology Medicine Database, China National Knowledge Database, Wan Fang Database, and Chongqing VIP Database. All case-control studies and cohort studies evaluating the relationship between the expression of Th17/Treg cells in peripheral blood and CSU will be searched. The search time limits from the establishment of the database to December 2020. The searches will be carried out using the Medical Subject Headings and all synonyms for Chronic Spontaneous Urticaria, Th17, Treg, case-control studies, and cohort studies.

### 2.5 Data extraction

Two authors (QY and WL) will independently extract data from the included studies. We will resolve discrepancies through discussion with a third researcher (MC). Data abstracted will include the first author, title, year of publication, sample size, study design, diagnostic criteria, and outcome measurement data.

### 2.6 Data synthesis and analysis

We will use RevMan V.5.3 (Copenhagen, The Nordic Cochrane Centre, The Cochrane Collaboration, 2014) statistical software for meta-analysis. The standardized mean difference will be used for continuous variables, and the odds ratio will be used for dichotomous data. The 95% confidence interval will be given for the results of each outcome indicator.

#### 2.6.1 Measures for heterogeneity

The chi-square test will be used to evaluate the statistical heterogeneity of the result of each study (*a* = 0.1), and *I*^2^ will be used to quantitatively judge the size of heterogeneity. If the *I*^2^ < 50% and *P* > .1, the fixed-effects model will be used for meta-analysis. If the *I*^2^ > 50% and *P* ≤ .1, the random-effects model will be used for meta-analysis. The obvious clinical heterogeneity will be treated by subgroup analysis, sensitivity analysis, or descriptive analysis.

#### 2.6.2 Subgroup analysis

Subgroup analysis will be performed based on the study types, the detection methods of Th17 and Treg cells.

#### 2.6.3 Sensitivity analysis

We will use the sensitivity analysis to check the reliability and stability of the outcome data. The weak studies will be removed from the robust studies, and meta-analysis will be conducted again to determine whether there is a significant difference in the effect size before and after the removal of the weak studies.

#### 2.6.4 Reporting bias

If there are sufficient studies, the funnel plot will be applied to check the reporting bias.

### 2.7 Quality assessment

The quality of each study will be evaluated by the Newcastle-Ottawa scale.^[14]^ Each study could obtain a maximum of 9 points,^[15]^ which categorized into 3 groups: very high risk of bias (0–3), high risk of bias (4–6), and low risk of bias (7–9).^[16]^

## 3 Discussion

Several studies had paid attention to the Th17/Treg imbalance in peripheral blood of patients with CSU.^[17–20]^ While a few articles had summarized the existing evidence. In this study, a comprehensive literature search will be conducted to systematically evaluate the relationship between the expression levels of Th17/Treg cells in peripheral blood and the pathogenesis of CSU. The results of the present study will summarize the latest evidence of the relationship between the Th17/Treg balance in peripheral blood and CSU. This study will not only provide helpful evidence for both patients and clinicians but also provides a theoretical basis for the pathogenesis of CSU.

However, there may be several limitations to this systemic review. First, the included studies and sample size are limited, which may affect the accuracy of the results. Second, this systematic review only searches the literature in Chinese and English, so the results are not globally representative. Third, this study excludes non-Chinese and English literature, there may be reporting bias. Fourth, we will include 2 types of studies, which may lead to heterogeneity of the results. We hope that this systematic review and meta-analysis can provide reliable evidence for the association between the Th17/Treg balance in peripheral blood and CSU.

## Author contributions

**Conceptualization:** Qianying Yu, Min Xiao, and Mingling Chen.

**Investigation:** Qianying Yu and Wenxia Lin.

**Supervision:** Wenxia Lin, Min Xiao, and Mingling Chen.

**Writing – original draft:** Qiaoqiao Kong, Rubin Zhong, and Yan Lan.

**Writing – review & editing:** Jie Zhang and Li Peng.
